# Possible stimulation of anti-tumor immunity using repeated cold stress: a hypothesis

**DOI:** 10.1186/1750-9378-2-20

**Published:** 2007-11-13

**Authors:** Nikolai A Shevchuk, Sasa Radoja

**Affiliations:** 1 Department of Radiation Oncology, Virginia Commonwealth University, Richmond, VA, USA; 2 Center for Cancer and Immunology Research, Children's Research Institute, Washington DC, USA

## Abstract

**Background:**

The phenomenon of hormesis, whereby small amounts of seemingly harmful or stressful agents can be beneficial for the health and lifespan of laboratory animals has been reported in literature. In particular, there is accumulating evidence that daily brief cold stress can increase both numbers and activity of peripheral cytotoxic T lymphocytes and natural killer cells, the major effectors of adaptive and innate tumor immunity, respectively. This type of regimen (for 8 days) has been shown to improve survival of mice infected with intracellular parasite *Toxoplasma gondii*, which would also be consistent with enhanced cell-mediated immunity.

**Presentation of the hypothesis:**

This paper hypothesizes that brief cold-water stress repeated daily over many months could enhance anti-tumor immunity and improve survival rate of a non-lymphoid cancer. The possible mechanism of the non-specific stimulation of cellular immunity by repeated cold stress appears to involve transient activation of the sympathetic nervous system, hypothalamic-pituitary-adrenal and hypothalamic-pituitary-thyroid axes, as described in more detail in the text. Daily moderate cold hydrotherapy is known to reduce pain and does not appear to have noticeable adverse effects on normal test subjects, although some studies have shown that it can cause transient arrhythmias in patients with heart problems and can also inhibit humoral immunity. Sudden immersion in ice-cold water can cause transient pulmonary edema and increase permeability of the blood-brain barrier, thereby increasing mortality of neurovirulent infections.

**Testing the hypothesis:**

The proposed procedure is an adapted cold swim (5–7 minutes at 20 degrees Celsius, includes gradual adaptation) to be tested on a mouse tumor model. Mortality, tumor size, and measurements of cellular immunity (numbers and activity of peripheral CD8+ T lymphocytes and natural killer cells) of the cold-exposed group would be compared to those of control groups (warm swim and no treatment). Cold-water stress would be administered twice a day for the duration of several months.

**Implications of the hypothesis:**

If the hypothesis is supported by empirical studies and the method is shown to be safe, this could lead to the development of an adjunctive immunotherapy for some (non-lymphoid) cancers, including those caused by viral infections.

## Background

Numerous studies show that small amounts of harmful or stressful agents (e.g. heat stress, cold stress, hypergravity) can be beneficial for the health of laboratory animals, the phenomenon that became known as hormesis, although evidence for possible benefits in humans is lacking at present [1,2]. This paper presents theoretical evidence for immunomodulating properties of brief cold stress, as increasing evidence indicates that cold stress repeated daily can have a stimulating effect on cell-mediated immunity [3-6], while inhibiting humoral immunity to some extent [7,8]. There is a number of ways to administer cold stress and this may account for the different effects on the immune system reported by various studies [9-11]. The focus of this paper is brief whole-body exposure to cold water since it has been shown to increase both activity and numbers of peripheral natural killer (NK) cells and CD8+ T lymphocytes [3,6,12-14]. This effect could be explained by transient activation of the sympathetic nervous system (SNS) [15,16], the hypothalamic-pituitary-adrenal (HPA) axis [17,18] as well as the hypothalamic-pituitary-thyroid (HPT) axis [19,20] resulting in a brief action of norepinephrine, adrenocorticotropic hormone (ACTH), beta-endorphin, and thyroid hormones (triiodothyronine (T3) and thyroxine (T4)) on cytotoxic T lymphocytes (CTLs) and NK cells. As explained below, all of these neuroendocrine factors have been previously shown to promote expansion and/or cytolytic activity of CTLs and NK cells. Interestingly, brief cold swim stress repeated for 8 days was also reported to increase survival of mice infected with intracellular parasite *Toxoplasma gondii *[5], the situation that is consistent with enhancement of cellular immunity. Based on this evidence and given the fact that NK cells and CTLs are major components of an anti-tumor immune response [21], it seems logical to propose the following hypothesis.

## Presentation of the hypothesis

The hypothesis is that brief cold-water stress repeated daily over many months could enhance anti-tumor immunity and improve cancer survival rate in a mouse (non-lymphoid) tumor model. While substantial efforts in the field of tumor immunology are devoted to finding ways to elicit a tumor-specific CTL response using tumor-derived antigens [22], this paper discusses a somewhat different approach of non-specific promotion of cell-mediated immune responses by increasing the total number and enhancing cytolytic potential of peripheral CTLs and NK cells. As a tide that lifts all boats, this approach could enhance endogenous (but possibly inadequate) CTL anti-tumor responses and thereby inhibit the development and growth of a tumor. Lymphocyte activation may also lead to autoimmunity, which does not appear to be the case for immunological changes associated with repeated cold stress [3,6,12,23,24]. A similar "broad activation" principle serves as a rationale for systemic interleukin-2 (IL-2) administration, which is currently used as anti-cancer immunotherapy in renal cell cancer and metastatic melanoma [25]. However, this approach proved to be rather inefficient, probably due to induction of T cell apoptosis and/or inability of the effector CD8+ T lymphocytes to infiltrate/home to the tumor site [25,26]. On the other hand, it has been demonstrated in several mouse tumor models that tumor-specific T cells are fully mature/activated and effectively infiltrate tumors but are not able to kill the cognate tumor cells *ex vivo *due to defective granule exocytosis-mediated cytotoxicity [27,28]. This defect is reversible as tumor-specific T cells that are isolated/purified from the tumor can regain lytic activity after brief *in vitro *culture [28]. The paper presented here hypothesizes that repeated cold stress could induce/enhance lytic activity of CD8+ T cells at the tumor site, and may also enhance innate anti-tumor immunity through expansion and activation of NK cells, all of which could result in more efficient tumor elimination. The detailed supporting evidence for this hypothesis is as follows.

### General neuro-endocrine effects of brief cold stress

From available literature, one can distinguish immediate (or transient) effects of cold stress, lasting 1 to 2 hours, and longer-term (or sustained) effects of repeated (e.g. once daily) cold stress, lasting days and possibly weeks. The immediate/transient effects reported in mice and humans include: brief activation of the SNS [15,16], of the HPA axis [17,18] and of the HPT axis [19,20] with a significant increase in the metabolic rate [29] and in plasma levels of norepinephrine [15,16], ACTH [30,31], corticosterone [7], beta-endorphin [18,32], T3 [19,20], T4 [20], as well as a modest or undetectable increase in the plasma levels of interleukin-6 (IL-6) [12,13] and cortisol [33,34] (Table 1). The sustained/longer-term effects of cold stress repeated daily or almost daily (over the period of 5 days to 6 weeks) appear to include increased plasma levels of tumor necrosis factor-α (TNF-α) [12], IL-2 [3], IL-6 [12], ACTH [35], corticosterone [3], as well as a decreased plasma level of α1-antitrypsin [12] and testosterone [6] (Table 1).

**Table 1:** Known effects of cold stress. "Immediate/transient" means effects of a single brief exposure to cold that last less than 1–2 hours. "Sustained/longer-term" means effects (of repeated daily or several times per week cold-water stress) that can last one day or longer.

**Immediate/transient effects**	**Sustained/longer-term effects**
*Activation of:* sympathetic nervous system [15,16] hypothalamic-pituitary-adrenal axis [17,18] hypothalamic-pituitary-thyroid axis [19,20] Increased resting metabolic rate [29]	*An increase in plasma levels of:* adrenocorticotropic hormone [35] corticosterone* [3] interleukin-2 (modest/undetectable) [3,84] interleukin-6 [12] tumor necrosis factor-α [12] haptoglobin [12] hemopexin [12]
*An increase in plasma levels of:* adrenocorticotropic hormone [30,31] norepinephrine [15,16] beta-endorphin [18,32] corticosterone* [7] cortisol (modest/undetectable) [33,34,82,83] thyroid stimulating hormone [20] triiodothyronine [19,20] thyroxine [20] interleukin-6 [6,13] Decreased plasma level of interleukin-2 [84,85]	*Decreased plasma levels of:* α1-antitrypsin [12] testosterone [6]

* It is expected to have a negligible immunosuppressive effect [86,87].

### Effects on maturation and peripheral recruitment of CTLs

Brief cold stress such as a cold swim has been reported to increase the level of peripheral T lymphocytes both in a transient manner after a single treatment (CD8+ T lymphocytes [11]) and in a sustained manner, when treatments are repeated daily or thrice per week (both CD4+ and CD8+ lymphocytes [3,12]). This could be mediated by the transient action of norepinephrine on β2-adrenergic receptors of CD8+ T lymphocytes [36-38] as a result of the activation of the SNS [39-41], which innervates both primary and secondary lymphoid organs and can stimulate lymphocyte proliferation, maturation, and peripheral recruitment via norepinephrine release in a non-synaptic fashion (reviewed in [42]). Additionally, the cold stress-induced expansion of CTLs could be mediated by changes in other neuroendocrine factors that are known to be caused by cold stress (Table 2).

**Table 2:** Possible mechanisms underlying the stimulatory effects of repeated cold stress on cell-mediated immunity

	**CTLs and other cytotoxic immune cells**	NK cells*
Expansion/peripheral recruitment	brief action** of the SNS [39-41] (via norepinephrine) on β2-adrenergic receptors of CD8+ T lymphocytes [36-38]; elevated plasma levels of ACTH [88,89], T4 [90], and beta-endorphin [91];	brief action** of the SNS (via norepinephrine) on β2-adrenergic receptors of NK cells [37,42,44]; upregulation of beta-endorphin [92], IL-2 [93], T3 [94] and T4 [95];
Activation	brief action** of the SNS (via norepinephrine) [36,39,96-98], beta-endorphin [91,99-101], T3 [102], or T4 [90,103,104] or sustained action of ACTH [88]; increased phagocytic index [43], downregulation of testosterone [105-107] or α1-antitrypsin [108];	-
Cytolytic activity	upregulation of ACTH [88,89], beta-endorphin [109,110], IL-2 [111-113], IL-6 [114,115], TNF-α [116-118], combination of IL-2 + IL-6 [119,120], or IL-2 + TNF-α [121]; downregulation of α1-antitrypsin [122,123];	elevated plasma levels of IL-2 [93], beta-endorphin [124-126], ACTH [127,128], T3 [129,130] or T4 [131,132];

* Continuous hypothermia [133] (lowered core body temperature which cannot normally result from a brief exposure to cold in most mammals [52,53]) or continuous exposure to acute cold (4 hours or longer [134]) have been shown to reduce both the number and activity of peripheral NK cells.

** Continuous elevated levels of norepinephrine or other β2-adrenergic agonists (3 hours or longer) appear to have an opposite effect: reduction in peripheral CTLs and NK cells and inhibition of their activity [42,135,136].

### Effects on activity of CTLs

Studies show that both single and repeated (daily) brief exposure to cold can enhance antigen-induced proliferative responses of T lymphocytes and the level of activated peripheral T lymphocytes in mammals [3,4,6,12,14]. This stimulatory effect could be mediated by the *brief *action of the SNS (via norepinephrine), of beta-endorphin, T3, or T4 or sustained action of ACTH and IL-2 (Table 2). There is also evidence of an increased phagocytic index following brief cold stress [43], suggesting that antigen (cross-)presentation may be enhanced. Unfortunately, it is not known if cold stress can enhance cytolytic activity of peripheral cytotoxic T lymphocytes, but there is indirect evidence that this may be the case (Table 2 and [5,24]).

### Effects on maturation and peripheral recruitment of NK cells

Single brief exposure to cold appears to transiently increase the level of peripheral NK cells [11,13]. This could be due to the increased peripheral recruitment and/or expansion of these cells, which are known to be mediated by the action of the SNS (via norepinephrine) on β2-adrenergic receptors of NK cells [37,42,44]. This effect could also be mediated by elevated levels of beta-endorphin, IL-2, T3 and T4 (Table 2).

### Effects on activity of NK cells

Both single and repeated (daily) brief cold stress has been shown to enhance cytolytic activity of NK cells [3,13]. This could be the result of elevated plasma levels of IL-2, beta-endorphin, ACTH, T3 or T4 (Table 2).

### Other effects

Daily moderate cold water stress does not appear to have noticeable adverse effects on normal test subjects either short-term or long-term [12,15,24,45] and, interestingly, in a near-life-time experiment on healthy rats, where the animals had to stand in 23°C water 4 hours per day 5 days per week, the repeated cold stress extended the lifespan by statistically insignificant 5% and somewhat reduced spontaneous incidence of tumors, especially sarcomas [24]. Based on the evidence presented earlier, more frequent exposure to cold of shorter duration, for example 5-minute cold swim stress twice per day (>7 hours apart), could have a more significant immunostimulatory effect and a less pronounced effect on metabolism compared to the above experiment. Cold water stress is also known to have an analgesic effect [32,46,47], which is often relevant in cancer.

### Possible adverse effects

1) Water colder than 14°C can cause cutaneous pain [48,49] and therefore would be best avoided.

2) Exposure to acute cold for extended periods of time can cause a significant drop of core body temperature (hypothermia) which can be associated with such adverse effects on health as ataxia, hypovolemia, atrial dysrhythmias, cold diuresis, and mental confusion [50,51]. Immersion in moderately cold water in the range of 16–23°C does not appear to cause hypothermia (core temperature of 35°C or lower) in healthy human subjects, even when it lasts for several hours [52]. During this procedure, core body temperature stays virtually unchanged during the first hour [52] due to unusual efficiency of the human thermoregulatory system [53]. It should be noted that the elderly or people with certain metabolic disorders may develop hypothermia under these conditions, and thus body temperature should be monitored in these groups of people if they use cold hydrotherapy [50,51].

3) Coldest months of the year have been shown to be associated with a higher incidence of acute heart failure and stroke [54-56]. In the landmark experiment by Holloszy and Smith described above, where rats were standing in cold water 4 hours per day, the prevalence of cardiovascular problems was increased according to postmortem examination, although the average lifespan was increased insignificantly and the prevalence of malignancies declined [24]. It is not known if daily *brief *exposure to moderately cold water (20°C, under 15 minutes) with gradual adaptation will have similar cardiovascular effects in the long run. Studies also show that immersion in cold water can cause transient arrhythmias in some patients with heart problems [57-59], although short-term cardiovascular effects of cold water immersion seem to be benign in normal test subjects [15].

4) Upper respiratory tract infections such as the flu and common cold occur predominantly although not exclusively during the cold time of the year, i.e. late fall/winter [60]. It is not known if this is due to exposure to cold, dietary patterns, or some other factors [61]. Near-life-time exposure of rats to cold water (23°C) for several hours per day was not reported to increase incidence of respiratory infections [24], as was the case for 1-hour cold water immersions (14°C) repeated 3 times per week for 6 weeks in humans [12,15]. On the other hand, some studies show that inhalation of cold air can inhibit cell-mediated immunity in the mucosa of the respiratory tract and thus possibly increase susceptibility to respiratory viral infections [62].

5) Swimming in ice-cold water can cause transient pulmonary edema in humans [63-65], which may be due to severe hypothermia among other things [51].

6) Some studies show that sudden immersion in ice-cold water can increase permeability of the blood-brain barrier in mice [66,67], and when repeated daily can also increase mortality of neurovirulent infections (e.g. West Nile virus, Sindbis virus) due to propagation of the infection to the brain of the mice [68,69]. Increased permeability of the blood-brain barrier can be due to the distress associated with a dive into ice-cold water because a different treatment which also causes distress to the animals, namely, isolation, has very similar adverse effects [67]. In addition, immersion in ice-cold water can rapidly cause severe hypothermia in mice [70] and hypothermia is known to increase permeability of the blood brain barrier in healthy animals [71]. The proposed adapted cold swim procedure at 20°C is not expected to compromise the blood-brain barrier in mice because it is designed to be minimally stressful (contains a gradual adaptation phase) and is brief, such that the core body temperature of the animals would remain above 35°C. The methods described in the "Testing the hypothesis" section could verify this assumption.

In summary, the review of literature suggests that winter swimming (in other words, sudden immersion in ice-cold water) may pose serious risks to health and it is possible that exposure to cold can be safer if it is brief and does not involve psychological distress, inhalation of cold air, and hypothermia. Further studies would be needed to assess the safety of the relatively non-stressful regimen of cold hydrotherapy that is proposed in this paper. At the same time, minimally stressful exposure to moderate cold still seems to have significant physiological and immunological effects [6,13,33,34], which may be useful for enhancing anti-tumor immunity according to our hypothesis.

## Testing the hypothesis

The hypothesis can be tested using a treatment that consists of an adapted cold swim procedure which begins with a slow and gradual lowering of a cage with laboratory animals into stirred cold water (20°C) over the period of 4–5 minutes followed by a 1- or 2-minute cold swim. The adaptation phase is expected to minimize stress and discomfort for the animals. The cage would be lifted out of the water to discontinue cold exposure and placed on proper bedding (certain cooling may still occur due to subsequent evaporation of water from the fur of the animals). The procedure is estimated to cause the core body temperature of mice to decline by approximately 2°C based on data from Wan R. *et al*. [70], and may have to be tested and optimized in order to avoid inducing significant hypothermia in the animals. Manual handling of individual mice would be best avoided. Cold water stress would be administered twice per day (more than 7 hours apart) according to a schedule described below. A syngeneic orthotopic mouse tumor model can be tested, for example, intradermal injection of MEB4 melanoma cell line (4 × 10^4 ^cells) in C57BL/6 mice [72] or subcutaneous injection of 6-1 tumor cell line (2 × 10^5 ^cells) in C3H/HeN mice [73]. Sixty mice can be used for a single experiment and all of them would be injected with tumor cells. One third of the animals will be assigned to the cold swim group. The other mice will be divided into two control groups: a warm swim group (Control I) and the no treatment group (Control II). Control I group will undergo adapted warm swim procedures (water temperature is 37°C) simultaneously with the cold swim (experimental) group. Control II group will undergo handling of the cage (and, possibly, manual handling of animals) similar to the experimental and Control I groups, but without immersion in water. Cold water stress would be initiated 2 weeks prior to injection of tumor cells and would be stopped for 3 days immediately after the inoculation in order to allow the skin wounds to close. Then cold water treatments would be resumed and continued for 2 months. On weekends, only one swim or, alternatively, no swims at all would be performed. At approximately 60 days postinjection, mortality, tumor incidence, and tumor size would be assessed in all three groups [72]. In addition, the numbers and cytolytic activity of peripheral NK cells [74-76] as well as average numbers of CD25-positive (activated) and total peripheral CD4+ T lymphocytes and CD8+ T lymphocytes [77,78] and their effector properties (i.e., TCR-induced cytokine production [79] and lytic function [28]) would be measured before initiation of cold water treatment (in 5–10 mice) and 60 days after the tumor inoculation in the experimental and control groups of mice. In a separate group of 10–20 tumor-injected mice, lytic activity of freshly isolated CD8+ tumor infiltrating (and peripheral) T lymphocytes would be assessed as a function of time after the initiation of cold stress treatment [28,73].

To verify if the proposed cold stress procedure does not significantly increase permeability of the blood-brain barrier in the animals, an Evans blue extravasation assay [80,81] can be performed in an additional small group of mice with a no-treatment control.

If the adapted cold swim is shown to be beneficial and safe in mouse tumor models, a similar regimen can be tested on human subjects, namely, adapted cold showers, 20°C, 2–5 minutes, preceded by a 5-minute gradual adaptation phase (expansion of the area of contact with water from the feet up), performed twice per day (morning and afternoon). The shower format is preferable in humans because the setup appears to take less time and effort compared to cold baths.

## Implications of the hypothesis

If the theory is confirmed by empirical studies and the proposed approach is shown to be safe, then some form of cold hydrotherapy could potentially become a treatment option for some (non-lymphoid) cancers as an adjunctive immunotherapy.

## Competing interests

The author(s) declare that they have no competing interests.

## Authors' contributions

Both NAS and SR contributed to formulating the idea and drafting of the manuscript. Both authors have read and approved the final manuscript.
